# Intracellular persistence of *Staphylococcus aureus* in endothelial cells is promoted by the absence of phenol-soluble modulins

**DOI:** 10.1080/21505594.2021.1910455

**Published:** 2021-04-12

**Authors:** Anke Siegmund, Muhammad Awais Afzal, Felix Tetzlaff, Daniela Keinhörster, Fabio Gratani, Kerstin Paprotka, Martin Westermann, Sandor Nietzsche, Christiane Wolz, Martin Fraunholz, Christian A. Hübner, Bettina Löffler, Lorena Tuchscherr

**Affiliations:** a Institute of Medical Microbiology, Jena University Hospital, Jena, Germany; b Institute of Human Genetics, Jena University Hospital, Friedrich Schiller Universität, Jena, Germany; c Interfaculty Institute for Microbiology and Infection Medicine Tübingen, Tübingen, Germany; d Biocenter, Chair of Microbiology, University of Würzburg, Würzburg, Germany; e Center for Electron Microscopy, Jena University Hospital, Jena, Germany

**Keywords:** *Staphylococcus aureus*, intracellular persistence, phenol-soluble modulins, lc3-vesicles, chronic infections

## Abstract

A large proportion of clinical *S. aureus* isolates that carry an inactive Agr system are associated with persistent infection that is difficult to treat. Once *S. aureus* is inside the bloodstream, it can cross the endothelial barrier and invade almost every organ in the human body. Endothelial cells can either be lysed by this pathogen or they serve as a niche for its intracellular long-term survival. Following phagocytosis, several vesicles such as phagosomes and autophagosomes, target intracellular *S. aureus* for elimination. *S. aureus* can escape from these vesicles into the host cytoplasm through the activation of phenol-soluble modulins (PSMs) αβ. Thereafter, it replicates and lyses the host cell to disseminate to adjacent tissues. Herein we demonstrate that staphylococcal strains which lack the expression of PSMs employ an alternative pathway to better persist within endothelial cells. The intracellular survival of *S. aureus* is associated with the co-localization of the autophagy marker LC3. In cell culture infection models, we found that the absence of *psmαβ* decreased the host cell lysis and increased staphylococcal long-term survival. This study explains the positive selection of *agr*-negative strains that lack the expression of *psmαβ* in chronic infection due to their advantage in surviving and evading the clearance system of the host.

## Introduction

*Staphylococcus aureus* (*S. aureus*) is a frequent pathogen that causes chronic and therapy-refractory infections, such as osteomyelitis and endocarditis [1–4]. Even though the bacteria are susceptible to antibiotics *in vitro*, antimicrobial therapy often fails to clear chronic infections, and surgical interventions, such as amputation, may become necessary [4]. *S. aureus* can act as a facultative intracellular pathogen capable of invading multiple types of host cells, such as endothelial cells and osteoblasts [5,6]. With its barrier function, endothelial cells have an important role in the human body. Once their integrity is compromised, adjacent tissues and the endothelium itself are more susceptible to infections [7,8]. The host cell invasion is coupled with the ability of *S. aureus* to persist in the intracellular environment for long-time periods. Intracellular staphylococcal adaptation is associated with phenotype switching to small colony variants (SCVs) [5]. SCVs are characterized by slow growth, reduced expression of virulence factors, and a reduced rate of metabolism [9–11]. Due to the reduced metabolism, antibiotics such as gentamicin and β-lactams are less effective in eliminating SCVs [12]. Furthermore, SCVs can hide inside the host cell without triggering a strong host response [13]. The signals, stress factors, and mechanisms that induce intracellular staphylococcal adaptation and phenotype switching remain unknown [5,9,14,15]. For many years, several studies have suggested a link between intracellular persistence and the stringent response for several pathogens [16] including *S. aureus* [17,18]. The stringent response can be induced by nutrient restriction and is characterized by an increased stress resistance of the bacterium. This response is initiated by rapid synthesis of the alarmones guanosine tetraphosphate and guanosine pentaphosphate (ppGpp and pppGpp) by the RSH, RelP, and RelQ enzymes. It was shown that the induction of the stringent response by phagocytized bacteria in human neutrophils leads to increased expression of cytotoxic phenol-soluble modulins (PSMs), which mediate escape after phagocytosis and bacterial survival in the cytoplasm [19]. PSMs belong to the family of amphipathic, α-helical peptides, and are important virulence factors of *S. aureus*. They can be classified into PSMα1-4, PSMβ1-2, and PSMδ. PSMα1-4 are smaller peptides with ~20-25 amino acids, whereas PSMβ1-2 are longer peptides with ~44 amino acids [20–23]. PSMs are positively regulated by Agr (accessory gene regulator, a global regulator related to quorum sensing) through direct binding of AgrA to their promoter [24] and during stringent response [19]. PSMs are involved in several processes, including cell death, biofilm formation, and phagosome escape [22,25]. Several studies have demonstrated that after phagocytosis, different intracellular vesicles can capture *S. aureus* for elimination [25–29]. *S. aureus* can escape from these vesicles to the cytosol for subsequent cytoplasmic replication in a PSM-dependent process [25]. In the cytoplasm, the bacteria can be sequestered in a double membrane-surrounded vesicle called autophagosome followed by elimination via a conserved intracellular degradation pathway called autophagy [30]. However, previous reports have described that *S. aureus* subverts or escapes autophagy by the expression of virulence factors transcriptionally regulated by Agr [29,31,32]. It was shown that *S. aureus* can escape from these vesicles to replicate in the cytosol, lyse the host cells, and disseminate to other tissues [31–34]. Although several studies reported the role of autophagy during *S. aureus* infection in epithelial cells such as HeLa and CHO cells [31,35,36], only a few studies focused on endothelial cells. Mice and endothelial cells with defects in the autophagic machinery turned out to be more susceptible to the infection by strong α-toxin producing *S. aureus* strains. Of note, Maurer *et al*. found that autophagy protects endothelial cells from the cytotoxic effect of α-toxin by a post-transcription modulation of the toxin receptor ADAM10 [37]. Also, the autophagy was impaired in the absence of the staphylococcal α-toxin [35,37]. Furthermore, the intracellular survival of *S. aureus* within endothelial cells was associated to autophagy [38]. Thus, the use of inhibitors of autophagy was suggested as a possible alternative to inhibit the intracellular survival of *S. aureus* in HeLa and Human Umbilical Vein Endothelial Cells (HUVECs) [39].

Surprisingly, a large proportion of clinical *S. aureus* isolates that are associated with severe persistent infections carry an inactivated Agr system [40,41]. In this work, we investigated the impact of the lack of *S. aureus* PSMs on intracellular persistence in endothelial cells (nonprofessional phagocytes). We demonstrate that due to the absence of *psmαβ S. aureus* resides within LC3^+^ vesicles, which leads to increase intracellular survival. Moreover, this intracellular pathway was exclusively observed in strains that lack the expression of *psmαβ* but no other Agr-regulated toxins such as α-toxin. This may explain the positive selection of *agr/psms*-negative strains during the development of chronic and difficult-to-treat infections.

## Materials and methods

### Bacterial strains

The *S. aureus* strains used in this study are listed in Table S1. The experiments carried out in this work were performed with the background strains LS1 and USA300 JE2. For testing the strains in the cell culture system, bacteria were grown overnight in brain-heart infusion (BHI) medium at 37°C with shaking (165 rpm). The following day, bacteria were adjusted to OD = 0.05 (578 nm) and incubated for 3 h at 37°C and 165 rpm until they reached the log phase. Exponential phase *S. aureus* was centrifuged at 5,000 rpm for 10 min and washed twice with sterile PBS 1X. The pellet was resuspended in fresh PBS and adjusted to OD = 1 (578 nm) for the infection assay.

### Generation of bacterial mutants

**Strains and plasmids**: The *S. aureus* strains USA300 JE2 and LS1 were used as the strain backgrounds to generate the mutants indicated under “Mutagenesis strategies” (Tables S1 and 2).

**Mutagenesis strategies**: The *relP, relQ,* and *rsh* mutants (*(p)ppGpp^0^*) of strains LS1 and USA300 were obtained by site-directed mutagenesis as previously described [42]. Mutagenesis vectors were transduced from RN4220 into the target strains. USA300 genes were mutated in the order *relP, relQ* and then *rsh* to generate the *(p)ppGpp^0^* strain. To generate the *(p)ppGpp^0^* strain in the LS1 background, the *rsh* synthase mutation was introduced first, followed by *relP* and *relQ* mutagenesis. All mutations were verified by PCR. *Psmα* and *psmβ* mutants were obtained by transducing the *psmα::tet(M)* and *psmβ::erm(C)* mutations into target strains using lysates of strains RN4220-307 and RN4220-308 [19].

### Cell culture

Human endothelial-like cells EA.hy926 (ATCC® CRL-2922™) were obtained from ATCC and tested to exclude *Mycoplasma* spp. contamination (PromoKine). Cells were maintained and grown in DMEM (PAN Biotech) with 10% fetal bovine serum (FBS) (Bio&Sell) and 1% HAT (Thermo Fisher). A confluence of 80% has been used for all experiments. Furthermore, the cells were prepared under confluent conditions [8].

### Flow cytometric cell death assay in EA.hy926 cells

To determine the cytotoxic effect of the LS1 and USA300 WT and mutant strains, EA.hy926 cells were plated at a concentration of 4 × 10^4^ cells/ml in 12-well plates (Greiner Bio-One) and incubated for 48 h until a confluence of 80% was observed. Next, the cells were washed with PBS (w/o Ca2+/Mg2+) and invasion medium (DMEM containing 1% HSA and 10 mM HEPES, pH 7.4) was added. Afterward, cells were infected with 50 µl (multiplicity of infection (MOI) = 180) of *S. aureus* adjusted to OD1 and incubated for 3 h at 37°C and 5% CO_2_. The used volume corresponded to an MOI of 180, since all strains grow with comparable kinetics (Fig. S1, Table S3). To remove all extracellular bacteria, the cells were treated with 20 µg/ml lysostaphin for 30 min. Fresh cell culture medium (DMEM containing 10% FBS, 1% HAT and 1% penicillin/streptomycin (Pen/Strep)) was added after washing and the cells were incubated for 24 h at 37°C and 5% CO_2_. Notably, Pen/Strep at the used concentration does not penetrate living cells and does not interfere with intracellular bacteria.

Dead cells in supernatant and adherent cells (detached with Trypsin/EDTA) were pooled from each sample and centrifuged for 5 min at 1,000 rpm. Next, the cells were carefully resuspended in PBS 1X and propidium iodide (PI) (50 µg/ml) was added. The dead cells were analyzed by flow cytometry (BD Accuri™ C6). Forward and sideward scatters (FSC-A and SSC-A) were used to identify the cell population of interest. Discrimination with FSC-H versus fluorescence (PE-H) was performed as a gating strategy to find the dead cells. PI fluorescence was measured using a 488-nm laser for excitation and a 585/540 nm laser filter for detection. A total of 5,000 events were recorded for each sample. Uninfected cells served as a negative control and cells treated with 70% EtOH were used as a positive control.

### Intracellular persistence assay in EA.hy926 cells

To determine the amount of intracellular bacteria, EA.hy926 cells were plated in 175 cm^2^ cell culture bottles (Greiner Bio-One). After 48 h of incubation the cells were washed with PBS (w/o Ca^2+^/Mg^2+^) and invasion medium (DMEM containing 1% HSA and 10 mM HEPES, pH 7.4) was added. Afterward, cells were infected with live bacteria at a MOI of 100. After 90 min of incubation, the cells were washed and treated with 20 µg/ml lysostaphin for 30 min to eradicate all adherent or extracellular staphylococci. Subsequently, fresh culture medium (DMEM containing 10% FBS, 1% HAT, and 1% Pen/Strep) was added to avoid bacterial overgrowth. Every two days, lysostaphin treatment (30 min) was repeated to kill all extracellular bacteria released from the cells. To determine the amount of intracellular bacteria cells were lysed with ice-cold H_2_O for 10 min at different time points (90 min, 2 days, and 7 days post infection (p.i.)). Serial dilutions of the lysates were plated on blood agar plates and incubated overnight at 37°C to determine the CFUs. Additionally, the number of WT and SCV-like colonies of the intracellular surviving bacteria was verified by a colony counter (Schuett colonyQuant). All colonies with a diameter <0.6 mm were considered SCVs. Due to the slow formation of SCVs, the final values of the amount of SCVs on agar were determined after 72 h of incubation. The amount of endothelial cells per time point was additionally counted to determine the CFU per cell.

### S. aureus *phagosomal escape assay by automated fluorescence microscopy*

Briefly, phagosomal escape of *S. aureus* expressing red fluorescent protein was microscopically detected in host cells stably expressing the fluorescent escape reporter YFP-CWT in the cytoplasm [42,43]. The cell wall-targeting domain (CWT) of the metallopeptidase lysostaphin shows strong affinity for the bacterial cell wall and is efficiently recruited to *S. aureus* upon translocation of the pathogen to the host cytosol. *S. aureus* strains were transduced with a plasmid expressing mRFPmars [44] under the control of the constitutive SarAP1 promoter. Hence, phagosomal escape was evident by YFP-CWT recruitment to red-fluorescent bacteria. DNA was stained with DAPI or Hoechst 34,850. Images were recorded with an Operetta System (PerkinElmer) using a 20x objective. Image analysis was performed with Harmony (PerkinElmer). The software identified host cell cytoplasm, nuclei, and spots in either green (YFP-CWT; escape) or red channels (mRFP; *S. aureus*). The mean relative escape rates (number of escape events as a fraction of all intracellular *S. aureus*) were scored in biological triplicates and technical duplicates.

### Co-localization of bacterial strains with LC3^+^ and LAMP-1^+^ membranes

To determine the co-localization of intracellular bacteria, EA.hy926 cells were plated on coverslips (Marienfeld) in 24-well cell culture plates (Greiner) following the protocol described for the intracellular persistence assay above. Cells were fixed on ice with ice-cold methanol for 10 min and permeabilized with 0.25% Triton X-100 for 10 min at RT at 3 h, 10 h, 24 h, and 48 h p.i. After blocking with 5% normal goat serum (NGS) in PBS 1X for 1 h, cells were co-stained with anti-mouse–LC3 (0260-100/LC3-2G6 nano Tools; 1:500), anti-rat LAMP-1 (Abcam, ab25245; 1:1000) and anti-rabbit *S. aureus* (Squarix, selfmade; 1:1000) antibodies overnight in blocking buffer. The next day, coverslips were washed with PBS 1X and incubated for 1 h with the corresponding secondary antibodies (Alexa Fluor 488: A11006 Thermo Fisher Scientific, Alexa Fluor 546: A-11,030 Thermo Fisher Scientific; Cyanine5: A10523 Thermo Fisher Scientific) in blocking buffer at RT. Then, coverslips were rinsed again with PBS 1X, stained with DAPI for 10 min and mounted in Fluoromount G (SouthernBiotech). Images were acquired with a Zeiss 880 confocal scanning fluorescence microscope. The number of LC3^+^ bacteria or LC3 and LAMP-1 double positive bacteria was assessed by co-localization plugin of ImageJ [45].

#### Transmission electron microscopy

EA.hy926 cells were grown and infected with *S. aureus* strains in 75 cm^2^ cell culture flasks. 24 h p.i. cells were washed with fresh medium and fixed with 2.5% v/v glutaraldehyde in protein-free medium for 2 h at room temperature. After washing 3 times for 15 min each with 0.1 M sodium cacodylate buffer (pH 7.2) the cells were post-fixed with 2% w/v osmiumtetroxide for 1 h at room temperature. After washing, the cells were scraped off and pelleted at 600 g. During the following dehydration in ascending ethanol series post-staining with 1% w/v uranylacetate was performed. Afterward, the pellets were embedded in epoxy resin (Araldite) and sectioned using a Leica Ultracut S (Leica, Wetzlar, Germany). Finally, ultrathin sections were mounted on filmed Cu grids, post-stained with lead citrate, and studied in a transmission electron microscope (EM 900, Zeiss, Oberkochen, Germany) at 80 kV. For image recording a 2 K slow scan CCD camera (TRS, Moorenweis, Germany) at a magnification of 20,000x was used.

### Statistical analysis

Analyses of data were conducted using GraphPad Prism 6.0 software (San Diego, CA). An unpaired t-test was used when two groups were compared. Multiple groups were compared by one-way ANOVA followed by Dunnett´s multiple comparisons test. According to the p-values, the differences were either not significant (ns, p > 0.05) or significant (* p < 0.05; ** p < 0.01; ***p < 0.001 and **** p < 0.0001).

## Results

### *Deletion of PSMαβ impairs the intracellular cytotoxicity of* S. aureus in vitro

**Figure 1.**
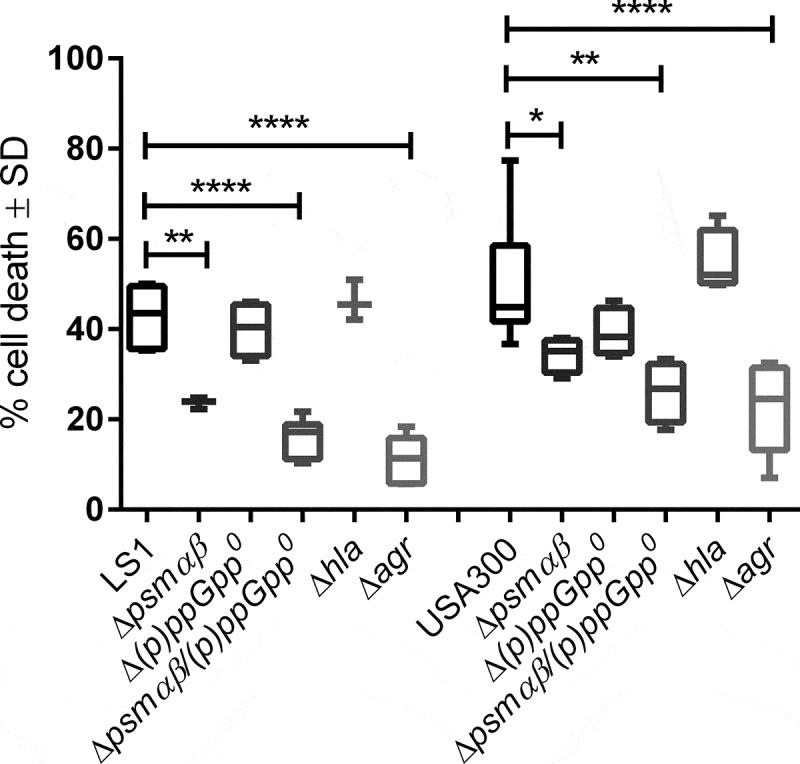
**The presence of PSMαβ is required to cause cell death on endothelial cells**. The cytotoxicity assays were performed in endothelial cells (EA.hy926) using LS1 and USA300 WT strains and the corresponding mutants. Cultured EA.hy926 cells were infected with *S. aureus* LS1 or USA300 or their derivate mutants. Cell death was measured after 24 h by flow cytometry using PI staining for dead cells. The values represent the means ± SD of four independent experiments. All mutant strains were compared to their corresponding WT by one-way ANOVA with Dunett multiple comparisons Test *p < 0.05; **p < 0.01, ***p < 0.001, ****p < 0.0001

*S. aureus* expresses a variety of cytotoxic virulence factors, such as the pore-forming α-toxin (*hla*), as well as membrane-active peptides, such as PSMs. PSM expression is transcriptionally driven by the quorum sensing system *agr* and by the stringent response [19,24]. To investigate the role of PSMs and their regulators in host cell death, we infected the endothelial-like cell line EA.hy926 for 3 h with *S. aureus* LS1, USA300 and isogenic mutants in factors involved in the stringent response (*(p)ppGpp^0^*), in *psmαβ* (Δ*psmαβ*), in *psmαβ* and the stringent response (Δ*psmαβ*/*(p)ppGpp^0^*), in α-toxin (Δ*hla)* and in *agr* expression (Δ*agr*) (Table S1). We characterized the growth curves of all strains but did not find differences between the mutant and parental strains (Fig. S1). Cytotoxicity was similar for the wild-type (WT) strains, stringent response mutants (*(p)ppGpp^0^*) and Δ*hla* mutants in both backgrounds. By contrast, the cytotoxicity of strains containing a deletion of *psmαβ* (Δ*psmαβ* and Δ*psmαβ*/*(p)ppGpp^0^*) or the corresponding regulator *agr* (Δ*agr)* was significantly reduced (Figure 1). These results indicate that cytotoxicity is impaired in the *S. aureus* strains that lack the expression of PSMs, suggesting that these mutants may contribute to maintain cell integrity required for persistence.

### *The lack of PSMαβ alters the intracellular persistence of* S. aureus in vitro

To persist inside host cells, *S. aureus* adheres to host structures, invades host cells, and develops different survival strategies to evade intracellular host defense system, such as SCV formation, in the chronic phase of infection [5,13]. To investigate the impact of PSMs and their regulators (Agr and the stringent response) on the intracellular persistence of *S. aureus*, EA.hy926 cells were infected with *S. aureus* LS1 and USA300 or their corresponding mutants (MOI = 100), and the number of recovered intracellular colony forming units (CFU) was analyzed up to 7 days p.i. Of note, the bacterial persistence was performed by using a lower MOI (100) in comparison to the cytotoxicity assay (Figure 1, MOI = 180) to prevent cell damage and evaluate the role of toxins in intracellular survival. Under these conditions, the viability of the cells was similar for all tested cells (Fig. S2).

**Figure 2.**
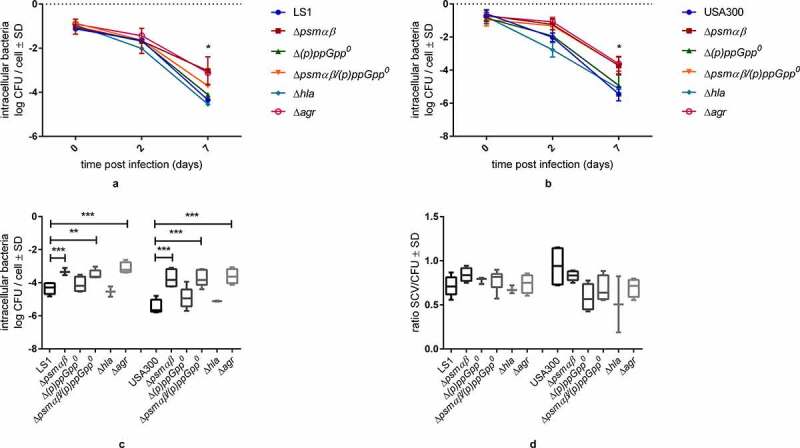
*psmαβ* expression has an impact on intracellular persistence in endothelial cells. Persistence assays were performed in EA.hy926 cells using *S. aureus* WT and mutant strains. Cultured EA.hy926 cells were infected with *S. aureus* strains LS1 and USA300 or their corresponding mutants (MOI 100) and infected cells were analyzed for up to 7 days. (a, b) The numbers of viable intracellular persisting bacteria per cell were determined on day 0, day 2 and day 7 p.i. by lysing host cells, plating the lysates on agar plates, and counting the colonies that have grown after 24 h and 48 h. (c) The log of intracellular CFU/cell recovered after 7 days post infection. (d) The Ratio of SCV/CFU on day 7. The values represent the means ± SD of five independent experiments. Significant differences in intracellular bacteria were detected for strains deficient in psmαβ and agr compared to their WT strain on day 7. The analysis was done by one-way ANOVA with Dunett multiple comparisons Test *p < 0.05; **p < 0.01, ***p < 0.001 ****p < 0.0001

All strains were internalized by EA.hy926 cells to the same extent (Fig. S3) and were able to persist at low numbers for both backgrounds for up to 7 days (Figure 2a and b). Whereas the *∆(p)ppGpp^0^* and *∆hla* strains were recovered with CFUs comparable to the WT strains, we observed significantly higher intracellular CFUs for the Δ*agr*, Δ*psmαβ* and Δ*psmαβ/(p)ppGpp^0^* strains 7 days p.i. (Figure 2a-C). No differences in the occurrence of SCVs on day 7 p.i. (Fig. S4) and the SCV/WT ratios among the parental and mutant strains were observed (Figure 2d), indicating that the formation of SCVs was not linked to the deletions of *psmαβ* and *agr*. Taken together, our results demonstrate that the lack of PSMαβ and no other Agr-dependent genes enhances the intracellular survival of *S. aureus* independent of the SCV formation.

### S. aureus *Δ*psmαβ *co-localize with LC3^+^ but not LAMP-1^+^ intracellular vesicles*

**Figure 3.**
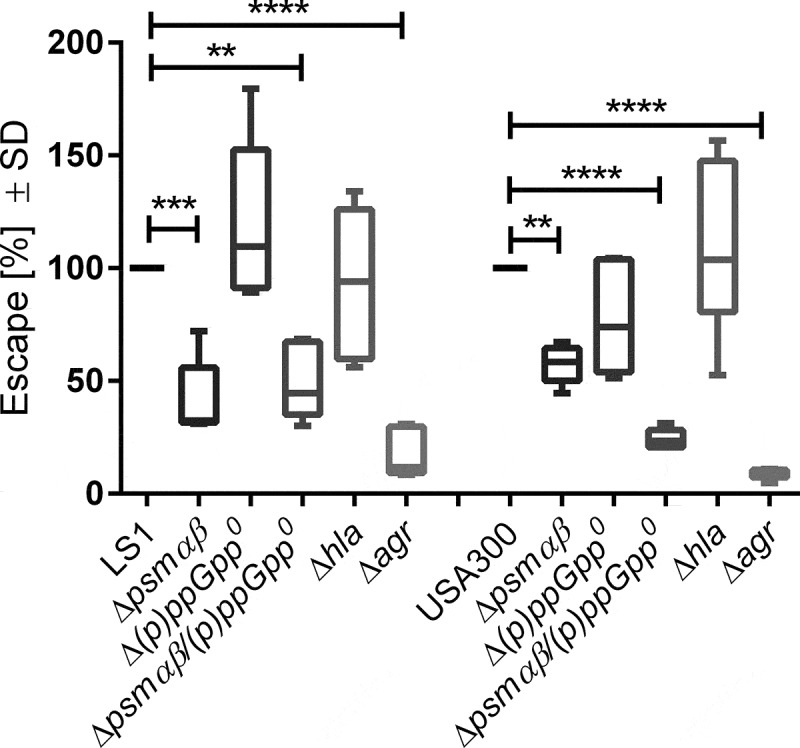
***psmαβ* expression has an impact on phagosomal escape in endothelial cells**. EA.hy926 YFP-CWT escape reporter cells were infected with LS1 and USA300 WT or their corresponding mutant strains at a multiplicity of infection of 10. Six hours p.i. samples were fixed and escape efficiencies of *S. aureus* strains LS1 and USA300 or their corresponding mutants were measured by fluorescence microscope. The values represent the means ± SD of six independent experiments, normalized to the WT strains. All mutant strains were compared to their corresponding WT by one-way ANOVA with Dunett multiple comparisons Test *p < 0.05; **p < 0.01, ***p < 0.001, ****p < 0.0001

After internalization by nonprofessional phagocytes, such as endothelial cells, *S. aureus* can escape from host vesicles and replicate in the cytoplasm [27] or is targeted by autophagy [46,47], and subversion of autophagy may contribute to the persistence of *S. aureus* within host cells. To test translocation of *S. aureus* to the host cytoplasm, we infected EA.hy926 cells expressing the fluorescent reporter YFP-CWT, which recognizes *S. aureus* in the host cytoplasm. We analyzed the phagosomal escape of mutants and parental *S. aureus* strains 6 h p.i. by enumerating YFP-CWT recruitment with an Operetta high-content imaging system (PerkinElmer) as previously published [43]. The escape of Δ*psmαβ*, Δ*psmαβ/(p)ppGpp^0^*, and Δ*agr* strains was dramatically reduced compared to the WT in both genetic backgrounds, LS1 and USA300. By contrast, the other analyzed strains did not show a reduction in phagosomal escape (Figure 3).

**Figure 4.**
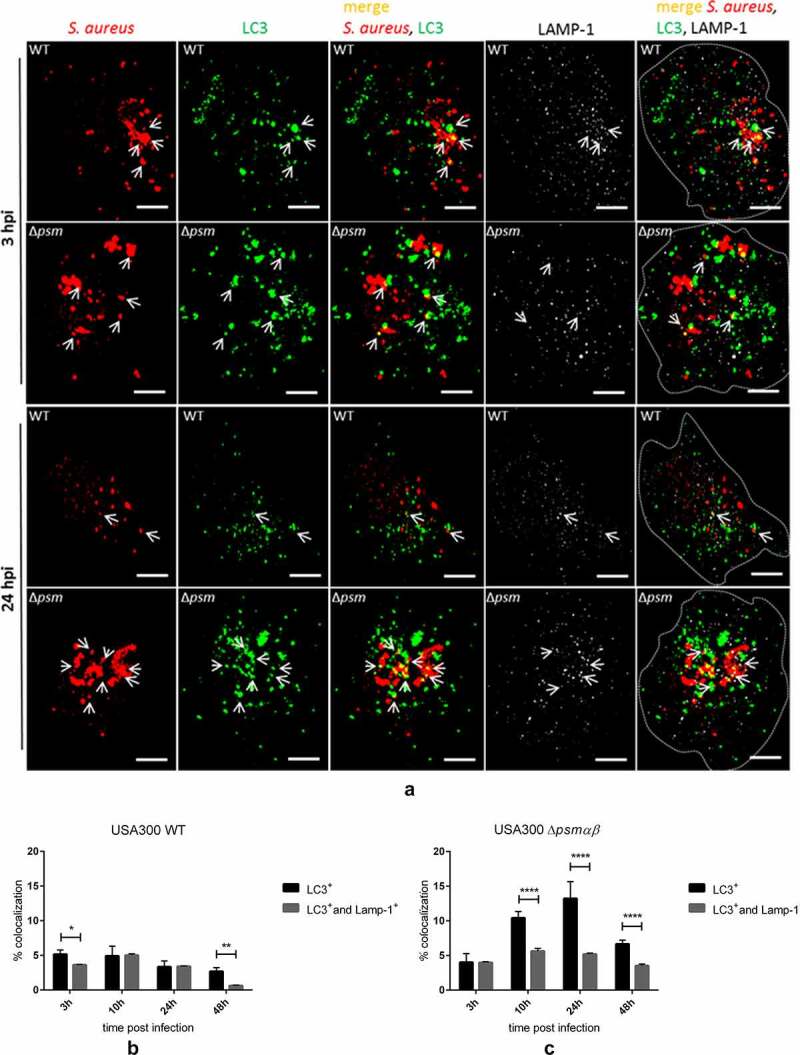
**Increased co-localization with autophagosomes and autolysosomes for the Δ*psmαβ* strain**. EA.hy926 cells were infected with USA300 WT and Δ*psmαβ*. On different time points p.i. (3 h, 10 h, 24 h and 48 h) cells were fixed and stained with autophagy marker LC3 and lysosome-associated membrane protein 1 (LAMP-1). (a) Confocal scanning fluorescence images are shown from representing time points 3 h and 24 h for both strains. Examples 10 h and 48 h p.i. are supplied in the supplementary information in Fig. S5. (b, c) Quantification of LC3 or LC3 and LAMP-1 associations with intracellular *S. aureus* WT or Δ*psmαβ* strains. Mean ± SD of *n* = 3 experiments (45 cells were analyzed per genotype at each time point); Bars: 5 µm; the mutant strain was compared to its corresponding WT by one-way ANOVA with Dunett multiple comparisons Test *p < 0.05; **p < 0.01, ***p < 0.001, ****p < 0.0001

Next, we followed the intracellular fate of WT *S. aureus* and the Δ*psmαβ* strain upon infection of EA.hy926 cells by immunostaining for the autophagy marker LC3 and lysosome-associated membrane protein 1 (LAMP-1) at different time points p.i. (Figure 4a-C). We observed a recruitment of LC3 and LAMP-1 to intracellular *S. aureus* WT (Figure 4a and b). We quantified the percentage of bacteria that co-localized with LC3 alone or LC3 and LAMP-1. The majority of *S. aureus* WT that were associated with LC3 also co-labeled with LAMP-1 (4B). In contrast, the majority of the *S. aureus* Δ*psmαβ* signals were associated with LC3 alone (4 C). These findings suggest that the mutant strain may reside for longer periods within LC3^+^ vesicles (Figure 4a-C). Of note, higher numbers of intracellular *S. aureus* Δ*psmαβ* were found in LC3^+^ vesicles with a peak at 24 h p.i. (Figure 4b-C) compared to the parental strain. After 48 h, WT and mutant strains were found significantly in LC3^+^ compared to LC3/LAMP-1^+^vesicles (Figure 4c and Fig. S5). These findings suggest that the lack of PSMs promotes the intracellular survival of *S. aureus* within LC3^+^ vesicles and may interfere with the recruitment of lysosomes.

**Figure 5.**
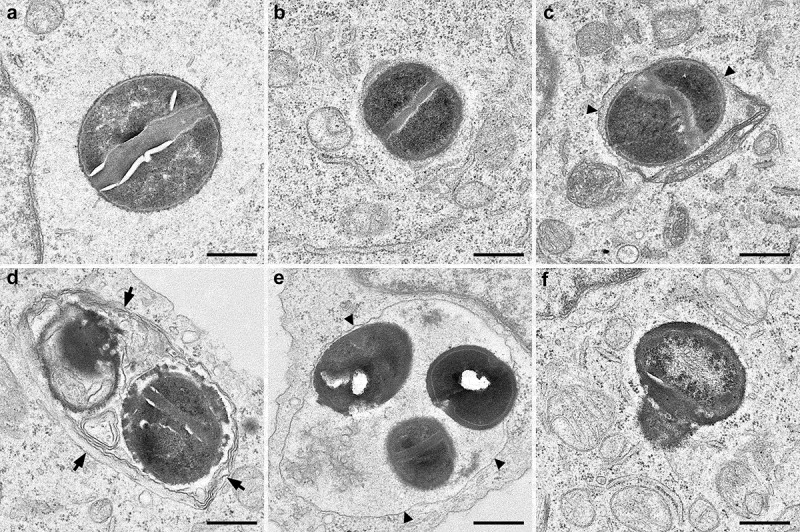
**Transmission electron microscopy images of intracellular *S. aureus* strains in cultured EA.hy926 cells 24 h p.i**. (a–c) USA300 WT located in the cytoplasm (a, b) or in a single-membrane vesicle (arrowheads, c). (d–f) Δ*psmαβ* located in multi-membrane vesicles (arrows, d), single-membrane vesicles (arrowheads, e), or in the cytoplasm (f). Of note, replicating bacteria were observed for both strains. Scale bars (a–f) are 500 nm. The small pure white areas are cracks in the ultrathin sections due to the harder substance

We next studied the localization of *S. aureus* WT and Δ*psmαβ* inside endothelial cells after 24 h p.i. by TEM (Figure 5). *S. aureus* WT was found mainly at the cytoplasm (Figure 5a, b) and some bacterial cells were surrounded by a single membrane as previously described (Figure 5c) [42]. In contrast, *S. aureus* Δ*psmαβ* was found mainly within vesicles (multi and single membranes; Figure 5d, e) and only few bacterial cells were located at the cytoplasm (figure 5f). Image analysis showed that both strains were alive and could actively replicate within vesicles (Figure 5).

## Discussion

*S. aureus* promotes its internalization and survival within host cells, and it is generally accepted that the pathogen thereby evades the cellular and humoral immune responses of the host. In addition, the intracellular bacterial location provides protection from the action of several antimicrobials [5,13,48]. Intracellular staphylococcal adaptation during infection is governed by cross-talk among global regulators that inhibit the expression of different virulence factors to promote survival within host cells [5,10,49,50]. Several authors have reported that staphylococcal strains isolated from patients who suffer from chronic infections lack the expression of *agr* and Agr-dependent virulence factors, such as PSMs [41,51,52]. Moreover, SCVs that are adapted for intracellular persistence have reduced expression of Agr [10]. Thus, the downregulation of *psmαβ* could represent an important bacterial strategy that contributes to the intracellular persistence of *S. aureus*. Recently, persisting *S. aureus* deficient in *psmαβ* was shown to be less susceptible for intracellularly acting antibiotics [53,54]. PSMs are transcriptionally controlled directly by Agr [24], and their expression is boosted during the stringent response [19].

After internalization by nonprofessional phagocytes, *S. aureus* is present in several types of vesicles, such as early and late endosomes, which eventually fuse with lysosomes. Autophagy is a host degradation system [30,55], that has also been shown to target intracellular pathogens in a process termed xenophagy [28], whereby pathogens of infected vesicles are targeted by the so-called autophagosome, which is decorated with LC3. Although autophagy was well studied in several nonprofessional cells, only few studies were performed with endothelial cells [38,56].

*S. aureus* has developed several strategies to survive in the intracellular environment by evading intracellular systems responsible for eliminating intracellular pathogens. PSMs, for instance, have been shown to be required for the escape of bacteria from endosomes [42]. *S. aureus* was further shown to manipulate autophagy in host cells [29,31,32,57]. However, the contribution of PSMs to escape from autophagosomes in endothelial cells is currently unclear.

We therefore investigated the *psmαβ*-dependent persistence of two different genetic backgrounds of *S. aureus* in a long-term endothelial infection model. By testing the cytotoxicity of *S. aureus* parental strains as well as mutants deficient in the production of PSMs, (p)ppGpp, and α-toxin, we found that PSMs are the main Agr-dependent factor involved in intracellular endothelial cell toxicity. Only strains lacking either expression of *psmαβ* or their main transcriptional regulator, *agr*, displayed a decrease in cell death when compared to that of the WT strains (Figure 1). Of note, *S. aureus* mutants in the stringent response or *hla* induced cell death at rates comparable to those of the respective parental strains. This effect was independent of cell entry, since none of the strains used differed significantly in host cell internalization (Fig. S3). Thus, our results suggest that the stringent response and α-toxin are not required for intracellular cytotoxicity of *S. aureus* and that the stringent response is not required for *psmαβ* expression. Our results indicate that strains that induce a reduced cell death rates may thus have higher chances for surviving within host cells for extended periods. Consequently, cell death *in vivo* may be manipulated by *S. aureus* to establish a persistent infection [58].

When we investigated the long-term intracellular survival of *S. aureus* in EA.hy926 cells, we observed that after 7 days, *S. aureus* mutants in *agr, psmαβ,* and *psmαβ*/*(p)ppGpp^0^* survived in higher numbers than the respective parental strains (Figure 2a-c). Since it was recently shown that *S. aureus agr* mutants reside in nonacidic vesicles and form a higher percentage of SCVs than WT strains [59]), we tested SCV formation in our model. We observed no differences in the formation of SCVs by *S. aureus* WT and ∆*psmαβ* strains (Figure 2d). These results imply that the surviving intracellular *S. aureus* population has a propensity to form SCVs but this phenotypic switch is independent of PSMαβ proficiency. Similarly, we can exclude the stringent response in accounting for intracellular survival within endothelial cells since we found that strains lacking crucial *(p)ppGpp* synthases did not show differences in long-term persistence (Figure 2a-C). The stringent response was linked to intracellular survival in professional phagocytic cells [19,60,61]) as a mechanism of stress defense of *S. aureus* and other bacteria [62,63] to address nutrition limitations. However, cell type-specific differences between phagocytes and tissue cells may be responsible for the observed differences.

The translocation of *S. aureus* from intracellular vesicles to the cytoplasm is described as a bacterial strategy to acquire nutrients, initiate bacterial replication, and evade antibacterial strategies of the host cells [25,27]. Accordingly, bacterial fitness is affected when the escape of *S. aureus* from intracellular vesicles is impaired and the pathogen resides for a prolonged period in these compartments characterized by poor nutrient content and bactericidal enzymes [42,64,65]. This “phagosomal escape” is predominantly achieved by the expression of PSMs [25,42,43,66]. However, the cytoplasmic replication of *S. aureus* is associated with subsequent host cell death [67], that terminates intracellular persistence of the bacteria. We therefore studied *S. aureus* translocation to the cytoplasm of infected EA.hy926 escape reporter cells and analyzed the role of important virulence factors and their regulators (Figure 3). We found that *S. aureus* USA300 and LS1 and their derivative mutants translocate to the cytoplasm of host cells. However, *agr* and *psmαβ* mutant strains escaped to a lesser extent and remained longer in intracellular vesicles. These results suggest that the strains, that do not escape phagosomes, induce less cell death, and thus survive in higher numbers within host cells (Figures 1, 2 and 3).

Some pathogens such as uropathogenic *Escherichia coli, Yersinia pseudotuberculosis,* and *Coxiella burnetii* actively use autophagy for their intracellular lifestyle [27]. *S. aureus* also undermines autophagy, but these processes seem to be host cell specific [28,31,46,55]. During autophagy, intracellular cytoplasmic bacteria are sequestered by LC3^+^ vesicles in a process that takes approximately 6 h [68]). Thus, we infected EA.hy926 cells and analyzed the co-localization of bacteria and LC3 as a marker for the activation of autophagy [26] and LAMP-1 as lysosome marker [35]). At 3 h p.i., the internalized bacteria co-localized with LC3^+^ vesicles for *S. aureus* WT and ∆*psmαβ* (Figure 4a-C), whereas LC3^+^ and LAMP-1 double positive vesicles were mainly associated to *S. aureus* WT (Figure 4b). In contrast, the majority of the vesicles occupied by *S. aureus* Δ*psmαβ* were associated only to LC3^+^ (Figure 4 C). These findings suggest that these strains may follow different pathways within endothelial cells. It is tempting to speculate that this early detection of LC3 co-localization with *S. aureus* may represent some kind of alternative intracellular pathway that involves this marker as well, so-called LC3-associated phagocytosis (LAP) described in professional phagocytes [26,28,69]). However, additional experimentation is required to investigate whether this pathway takes place in nonprofessional phagocytes. *S. aureus* ∆*psmαβ* was found to be associated with LC3^+^ vesicles in significantly higher numbers than the WT strain. This association lasted for up to at least 24 h p.i. and thus longer than the LC3-association of the parental strain (Figure 4a-C). However, the co-localization of *S. aureus* ∆*psmαβ* and LC3^+^ vesicles was not permanent, and a significant reduction was observed after 48 h p.i. (Fig. S5), suggesting that: (i) either other virulence factors may contribute to escape from intracellular vesicles to the cytoplasm at this stage or (ii) that LC3-association is subsequently lost from bacteria-containing vesicles or (iii) intracellular bacteria are localized in another vesicle at this time point.

**Figure 6.**
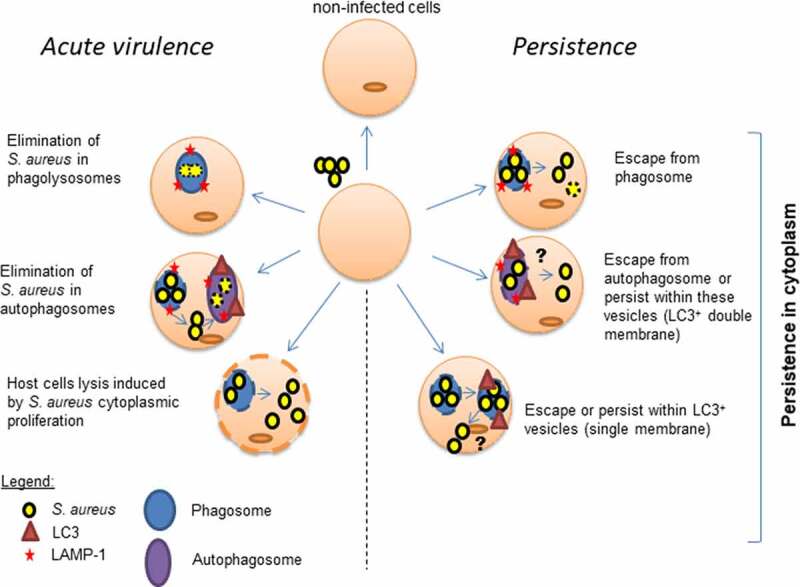
**Intracellular pathways of *S. aureus* within endothelial cells**. *S. aureus* is internalized by endothelial cells and several host-pathogen fates coexist which leads to induce the acute or chronic infection. Some cells are not infected by bacteria. *Acute virulence* pathways include the elimination of *S. aureus* within phagolysosomes or autophagoslysosomes. Host cell death is trigged by bacterial toxins expressed during the cytoplasmic proliferation of *S. aureus. Persistence* of *S. aureus* is characterized by the poor elimination of bacteria by host cells. *S. aureus* is able to escape from phagosomes and/or autophagosomes and persist in the cytoplasm. *S. aureus* that lacks the production of PSMs is able to persist in higher number within endothelial cells. This persistence may be related with the possibility to reside in LC3^+^ vesicles and avoid the recruitment of LAMP-1. LC3^+^ vesicles containing bacteria may be generated from phagosome or autophagosome. Partial damage of phagosome membrane may recruit LC3 and *S. aureus* may persist within single membrane compartment. Alternatively, *S. aureus* may persist within autophagosomes (LC3^+^ vesicles with double membrane). Further studies are necessary to determine whether *S. aureus* resides or escapes from LC3^+^ vesicles with single or double membrane. The bacterial survival in cytoplasm is enhanced by forming SCVs which allow the bacteria to persist under non-favorable conditions. However, the formation of SCVs occurs independently of the expression of PSMs

In conclusion, our study of the intracellular fate of two different backgrounds of staphylococcal strains and their mutants in PSMαβ suggests that *S. aureus* strains can persist in LC3^+^ vesicles in endothelial cells. The localization of *S. aureus* Δ*psmαβ* within these vesicles impairs the elimination of this pathogen by the host and instead promotes bacterial persistence and survival (Figure 6). Bacteria are internalized by endothelial cells and several host-pathogen fates coexist which leads to several acute or chronic infection courses and enhance bacterial survival (bed-hedging-strategy) [70–72] (Figs. 5, 6). Host cells can eliminate *S. aureus* within phagolysosomes or autophagolysosomes [35,42,57]. However, *S. aureus* can escape from phagosomes or autophagolysosomes, proliferate within the cytoplasm, and triggers the host cell lyses by secretion of toxins [58]. All these conditions do not lead to persistence. Intracellular persistence of *S. aureus* involves different pathways that may take place simultaneously upon bacterial internalization. *S. aureus* can escape from phagosomes [42] or autophagosomes [35,56] to cytoplasm and form SCVs to survive under the restricted intracellular conditions for prolonged periods within endothelial cells [5,10]. Another persisting pathway described in this study is the survival within LC3^+^ vesicles in PSM-downregulated/absent manner. The formation of LC3^+^ vesicles may be originated by two different pathways: from the phagosome (single membrane) or the autophagosome (double/multi-membrane) (Figure 5) that may not get acidified (Figure 4) [25]. Inside these vesicles, *S. aureus* can replicate (Figure 5) and may be protected from host intracellular defenses enhancing the persistence. Further investigation is needed to find out whether and how *S. aureus* may reside within these vesicles or escape and persist in the cytoplasm.

In this way, the endothelial cells represent a niche for the persistence of *S. aureus* within the host. Our work highlights the importance of studying attenuated *S. aureus* strains lacking the expression of *psmαβ* or *agr* since these strains are often encountered in a clinical setting and represent a potential health concern due to their ability to evade the immune system and antimicrobial activity. Further understanding of the different mechanisms of host–pathogen interactions may provide new targets for difficult-to-treat chronic infections.

## Supplementary Material

Supplemental Material
