# Supplementary material for: Intracellular persistence of Staphylococcus aureus in endothelial cells is promoted by the absence of phenol-soluble modulins
Source: Virulence. 2021 Apr 12;12(1):1186–98. doi: 10.1080/21505594.2021.1910455 (PMC8043190; doi:10.1080/21505594.2021.1910455)
Supplement: Supplemental Material [file KVIR_A_1910455_SM8248.docx]

**Intracellular persistence of *Staphylococcus aureus* in endothelial cells is promoted by the absence of phenol-soluble modulins**

Anke Siegmund^1^, Muhammad Awais Afzal^2^, Felix Tetzlaff^2^, Daniela Keinhörster^3^, Fabio Gratani^3^, Kerstin Paprotka^4^, Martin Westermann^5^, Sandor Nietzsche^5^, Christiane Wolz^3^, Martin Fraunholz^4^, Christian A. Hübner^2^, Bettina Löffler^1^, Lorena Tuchscherr^1*^

^1^ Institute of Medical Microbiology, Jena University Hospital, Jena, Germany; ^2^ Institute of Human Genetics, Jena University Hospital, Friedrich Schiller Universität, Jena, Germany; ^3^ Interfaculty Institute for Microbiology and Infection Medicine Tübingen, Tübingen, Germany; ^4^ Biocenter, Chair of Microbiology, University of Würzburg, Würzburg, Germany, ^5^ Center for Electron Microscopy, Jena University Hospital, Jena, Germany.

*[Lorena.Tuchscherrdehauschopp@med.uni-jena.de](mailto:Lorena.Tuchscherrdehauschopp@med.uni-jena.de)

**S1 Table: Strains and plasmids**

| **Strain name in the paper** | **Strain** | **Description** | **Source/ Reference** |
| --- | --- | --- | --- |
| ***E. coli*** |  |  |  |
| Top10 | Top10 | Competent *E. coli* plasmid transformation | Invitrogen |
| ***S. aureus*** |  |  |  |
| RN4220 | RN4220 | Restriction-deficient derivate of 8325-4, rK−mK+ | (1) |
| LS1 | LS1 | septic arthritis isolate | (2) |
| LS1 ∆*(p)ppGpp0* | LS1-86-306-230 | LS1 derivative; mutated *rsh_syn_* (Δ942-950nt), *ΔrelP*, mutated *relQ* synthase (Δ343-429nt) | This work |
| LS1 ∆*psmαβ* | LS1-307-308 | LS1 derivate; *psmα::tet(M)*, *psmß::erm(C)* | This work |
| LS1 ∆*psmαβ/(p)ppGpp0* | LS1-L1-307-308 | LS1 derivate; mutated *rsh* synthase (Δ942-950nt), *ΔrelP*, mutated *relQ* synthase (Δ343-429nt), *psmα1-4::tet(M)*, *psmß1-2::erm(C)* | This work |
| LS1 ∆*agr* | HOM150 | LS1 derivative carrying the *agr::tetM* deletion of RN6911; Tc^R^ | (3) |
| LS1 ∆*hla* | LS1∆*hla* | LS1 Δ*hla*, ErmR, obtained by phage transduction from DU1090 | (4) |
| USA300 | USA300 JE2 | Strain USA 300 LAC cured of all 3 native plasmids | (5) |
| USA300 ∆*(p)ppGpp0* | USA300-229-230-263 | JE2 mutated *relP* synthase (Δ450-536nt), mutated *relQ* synthase (Δ343-429nt), mutated *rsh* (Δ249-951nt) | This work |
| USA300 ∆*psmαβ* | USA300-307-308 | JE2 *psmα1-4::tet(M)*, *psmß1-2::erm(C)* | This work |
| USA300 ∆*psmαβ/(p)ppGpp0* | USA300-229-230-263-307-308 | JE2 mutated *relP* synthase (Δ450-536nt), mutated *relQ* synthase (Δ343-429nt), mutated *rsh* (Δ249-951nt), *psmα1-4::tet(M)*, *psmß1-2::erm(C)* | This work |
| USA300 ∆*agr* | USA300-391 | JE2 Δ*agr* | (4) |
| USA300 Δ*hla* | USA300 Δ*hla* | JE2 Δ*hla*:erm | (4) |
| **Plasmids** |  | **Description** | **Source/ Reference** |
| pBASE |  | tetracyclin inducible suicide mutagenesis vector | (5) |
| pCG86 |  | pKOR1 with mutated *rsh* synthase (Δ942-950nt) | (6) |
| pCG229 |  | pKOR1 with mutated *relP* synthase (Δ450-536nt) | (7) |
| pCG230 |  | pKOR1 with mutated *relQ* synthase (Δ343-429nt) | (7) |
| pCG263 |  | pKOR1 with mutated *rsh* (Δ249-951nt) | (7) |
| pCG306 |  | pBASE with vector with flanking regions of relP-locus | This work |
| pCG307 |  | pBASE vector with *psmα*1-4:: tet(M) replacement | (5) |
| pCG308 |  | pBASE vector with *psmβ*1-2::erm(C) replacement | (5) |
| pCG391 |  | pBASE6 vector with flanking regions of *agr*-locus | (4) |

**S2 Table: Oligonucleotides used for mutagenesis**

| **Purpose and Description** | **Template** | **Name** | **Sequence** |
| --- | --- | --- | --- |
| Generation of plasmid pCG306 | HG001 | \| relPdel-rev \| \| --- \| \| relPdel-for \| \| bglIIrelPdel-for \| \| salIrelPdel-rev \| | \| TATCGGAGGTTAGTATAAAA \| \| --- \| \| ATGTTTAGTAGGTGTGTTAC \| \| AAAAAGATCTAGATGTTGTTCCTATTGCGG \| \| AAAAGTCGACTAAATGAAGCGTCGACGTCT \| |
| Verification of Δ*relP* mutant | LS1-86-306-230 | \| bglIIrelPdel-for \| \| --- \| \| salIrelPdel-rev \| | \| AAAAAGATCTAGATGTTGTTCCTATTGCGG \| \| --- \| \| AAAAGTCGACTAAATGAAGCGTCGACGTCT \| |
| verification of *relP* synthase mutant | USA300-229-230-263; | relPDIG-for  relPDIG-rev | GTCGCACATTCTTTCAGT  CGTTATTAGGTTTCGTAGAGTT |
| verification of *relQ* synthase mutant | USA300-229-230-263;  LS1-86-306-230 | relQDIGfor2  relQDIGrev2 | TTCGTAACACTAAAGAAAGTGG  GCGTGTAATATTTTTGAGCT |
| verification of *rsh* mutant | USA300-229-230-263; | rel431for  relLC4rev | GCGTGGCTTTATCATTGG  ACTTCAACCATCATTCGG |
| verification of *rsh* synthase mutant | LS1-86-306-230 | rel1775for  relLC3rev | CGGCTCTTCGTTATATTGATAA  GGCAACTCAATAACATCAC |

Artificial restriction sites are underlined.





**A**

**B**





**Fig. S1: Growth curves** of *S. aureus* (A) LS1 and (B) USA300 WT and their corresponding mutant strains were performed in BHI medium of 6 h.

**Table S3:** **Generation time and the growing rates (µ).**

|  | **LS1 WT** | **Δ*psmαβ*** | **Δ*(p)ppGpp^0^*** | **Δ*psmαβ/(p)ppGpp^0^*** | **Δ*agr*** |
| --- | --- | --- | --- | --- | --- |
| **µ(h^-1^)** | 2.36 | 2.14 | 2.07 | 2.21 | 2.13 |
| **Generation time (min)** | 17.64 | 19.45 | 20.09 | 18.79 | 19.52 |
|  | **USA300 WT** | **Δ*psmαβ*** | **Δ*(p)ppGpp^0^*** | **Δ*psmαβ/(p)ppGpp^0^*** | **Δ*agr*** |
| **µ(h^-1^)** | 2.33 | 2.49 | 2.46 | 2.51 | 2.28 |
| **Generation time (min)** | 17.84 | 16.68 | 16.88 | 16.58 | 16.99 |

.





**A**

**B**





**Fig. S2: Cell viability during persistence.** (A, B) represent the viability of the infected endothelial cells during persistence. No differences between the WT or their corresponding mutant strains were observed. The values represent the means ± SD of three independent experiments. All mutant strains were compared to their corresponding WT by one-way ANOVA with Dunett multiple comparisons test.





**Fig. S3: Host cell invasion upon infection with LS1 and USA300 WT strains and their corresponding mutants.** Cultured EA.hy926 endothelial cells were infected with *S. aureus* strains LS1 and USA300 or their corresponding mutants (MOI 100). 90 min p.i. (day 0) the amount of intracellular bacteria were analyzed by lysing the cells and plating the intracellular bacteria on blood agar plates. The log of intracellular CFU per cell recovered 90 min p.i. are shown detected after 24 h and 48 h of incubation. The values represent the means ± SD of five independent experiments. All mutant strains were compared to their corresponding WT by one-way ANOVA with Dunett multiple comparisons test. No significant differences were observed.


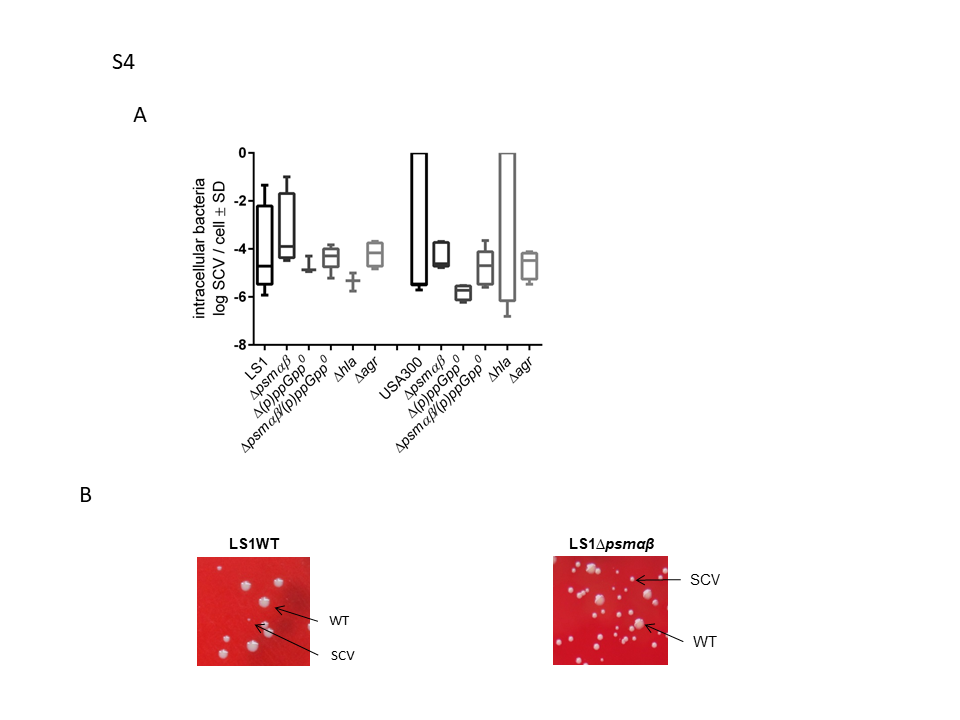


**Fig. S4:** (A) **The log of intracellular SCVs per cell recovered after 7 days post infection**. The values represent the means ±SD of four independent experiments. All mutant strains were compared to their corresponding WT by one-way ANOVA with Dunett multiple comparisons test *p < 0.05; **p < 0.01, ***p < 0.001 ****p<0.0001. (B) Pictures of *S. aureus* LS1 WT and LS1 Δ*psmαβ* strains lysed 7 days p.i. and plated on sheep blood agar plates for 72 h at 37°C and 5% CO_2_.


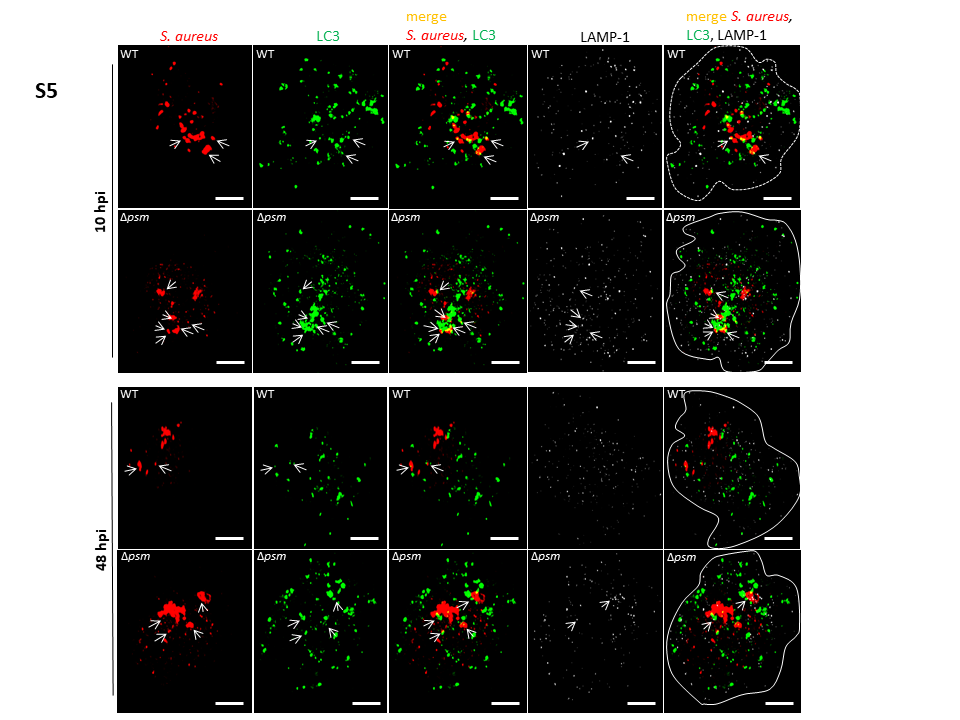


**Fig. S5: Increased co-localization with autophagosomes and autolysosomes for the Δ*psmαβ* strain.** EA.hy926 cells were infected with USA300 WT and Δ*psmαβ*. On different time points p.i. (here 10 h and 48 h p.i.) cells were fixed and stained with autophagy marker LC3 and lysosome-associated membrane protein 1 (LAMP-1); n=3 experiments (45 cells were analyzed per genotype at each time point); Bars: 5 µm.

1. Kreiswirth BN, Löfdahl S, Betley MJ, O'Reilly M, Schlievert PM, Bergdoll MS, et al. The toxic shock syndrome exotoxin structural gene is not detectably transmitted by a prophage. Nature. 1983;305(5936):709-12.

2. Tuchscherr L, Bischoff M, Lattar SM, Noto Llana M, Pfortner H, Niemann S, et al. Sigma Factor SigB Is Crucial to Mediate *Staphylococcus aureus* Adaptation during Chronic Infections. PLoS pathogens. 2015;11(4):e1004870.

3. Schmitt J, Joost I, Skaar EP, Herrmann M, Bischoff M. Haemin represses the haemolytic activity of *Staphylococcus aureus* in an Sae-dependent manner. Microbiology (Reading, England). 2012;158(Pt 10):2619-31.

4. Munzenmayer L, Geiger T, Daiber E, Schulte B, Autenrieth SE, Fraunholz M, et al. Influence of Sae-regulated and Agr-regulated factors on the escape of *Staphylococcus aureus* from human macrophages. Cellular microbiology. 2016;18(8):1172-83.

5. Geiger T, Francois P, Liebeke M, Fraunholz M, Goerke C, Krismer B, et al. The stringent response of *Staphylococcus aureus* and its impact on survival after phagocytosis through the induction of intracellular PSMs expression. PLoS pathogens. 2012;8(11):e1003016.

6. Geiger T, Goerke C, Fritz M, Schafer T, Ohlsen K, Liebeke M, et al. Role of the (p)ppGpp synthase RSH, a RelA/SpoT homolog, in stringent response and virulence of *Staphylococcus aureus*. Infection and immunity. 2010;78(5):1873-83.

7. Geiger T, Wolz C. Intersection of the stringent response and the CodY regulon in low GC Gram-positive bacteria. International journal of medical microbiology : IJMM. 2014;304(2):150-5.
